# Genetically Confirmed Osteogenesis Imperfecta (COL1A1) With Unexplained Ambiguous Genitalia in a 46,XY Child: An Index Case Report

**DOI:** 10.1002/ccr3.73016

**Published:** 2026-07-08

**Authors:** Harshita Agarwal, Tathagata Jha, Mohammad Orooj Azmi, Soumita Sarkar, Vinay Suresh

**Affiliations:** ^1^ Institute of Post Graduate Medical Education and Research Kolkata West Bengal India; ^2^ Medical College Kolkata West Bengal India; ^3^ University of Oxford Oxford UK

**Keywords:** ambiguous genitalia, case report, COL1A1, CYP11A1, disorders of sex development, osteogenesis imperfecta

## Abstract

Osteogenesis imperfecta (OI) is a heritable disorder of type I collagen characterized by bone fragility, blue sclerae, and short stature. Disorders of sex development (DSD) in 46,XY individuals demand comprehensive endocrine, imaging, and genetic evaluation. We report a 6‐year‐old child with recurrent low‐impact fractures, blue sclerae, short stature, and ambiguous genitalia. Karyotype was repeatedly reported as 46,XY. Subsequent endocrine evaluation revealed elevated follicle‐stimulating hormone (16.03 mIU/mL), low luteinizing hormone (1.35 mIU/mL), markedly reduced testosterone (< 7 ng/dL), low dehydroepiandrosterone sulfate (< 15 μg/dL), and androstenedione (< 0.300 ng/mL), consistent with primary gonadal dysgenesis. Congenital adrenal hyperplasia was provisionally excluded. Whole‐exome sequencing identified a heterozygous pathogenic frameshift variant in COL1A1 (c.441delC; p.Gly148fs*117), confirming OI, and a heterozygous CYP11A1 variant of uncertain significance (c.119 T > C; p.Ile40Thr), possibly contributing to partial adrenal insufficiency and sex reversal. The patient receives annual zoledronate, calcium, and vitamin D supplementation, with ongoing multidisciplinary follow‐up. This represents the first reported coexistence of OI due to COL1A1 mutation with ambiguous genitalia in a 46,XY child, emphasizing the complexity of overlapping skeletal and gonadal phenotypes and the importance of longitudinal care.

## Key Clinical Message

We report the first known association of osteogenesis imperfecta from a COL1A1 frameshift mutation coexisting with ambiguous genitalia in a 46,XY child. This index case illustrates the need for integrated genomic and endocrine evaluation in atypical skeletal dysplasia and expands the known phenotype spectrum of COL1A1‐associated conditions.

## Graphical abstract

A 6‐year‐old child with a rare dual diagnosis confirmed by genetic testing ‐ osteogenesis imperfecta (blue sclerae, disproportionate short stature) and concurrent 46,XY disorder of sex development (micropenis, hypospadias, impalpable gonads).

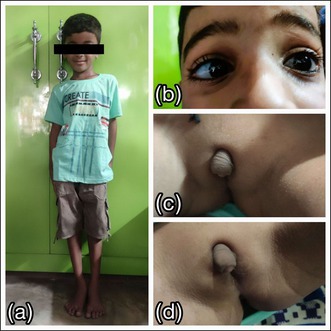

## 1 Introduction

Osteogenesis imperfecta (OI) is a genetically heterogeneous connective tissue disorder caused by mutations in collagen type I alpha 1 chain (COL1A1) or collagen type I alpha 2 chain (COL1A2), leading to abnormal type I collagen synthesis and consequent bone fragility [1]. Classical features include recurrent low‐impact fractures, blue sclerae, dentinogenesis imperfecta, and variable short stature [1].

Disorders of sex development (DSDs) are congenital conditions in which chromosomal, gonadal, or phenotypic sex is atypical [2]. In 46,XY DSD, common etiologies include defects of androgen biosynthesis or action, primary gonadal dysgenesis, persistent Müllerian duct syndrome (retention of female‐type internal genital structures in a genetic male due to impaired anti‐Müllerian hormone signaling), ovotesticular DSD (presence of both ovarian and testicular tissue in the same individual), and steroidogenic enzyme deficiencies such as CYP11A1/cytochrome P450 side‐chain cleavage enzyme (P450scc) deficiency [2, 3]. A multidisciplinary diagnostic approach, incorporating karyotype, endocrine evaluation, imaging, and targeted or genome‐wide genetic testing, is standard.

We report a 6‐year‐old child with genetically confirmed COL1A1‐related OI and lifelong ambiguous genitalia (46,XY), in whom sequential endocrine, radiological, and whole‐exome sequencing investigations did not identify a single unifying cause for the DSD. To our knowledge, the coexistence of a pathogenic COL1A1 variant with ambiguous genitalia in a patient with isolated OI has not been previously documented. A detailed chronological reconstruction of the clinical course and investigations is presented.

## 2 Case Presentation

### 2.1 Case History

We report on a 6‐year‐old child with a history of recurrent low‐impact fractures, blue sclerae, short stature, and lifelong ambiguous genitalia. The child was born at term via vaginal delivery, with birth weight documented as 2.7 kg, and no perinatal or postnatal complications were reported. Ambiguous genitalia were noted at birth, but no immediate intervention was undertaken. At 6 days of age, hormonal assays revealed dehydroepiandrosterone sulfate (DHEAS) of 122.9 μg/dL (normal neonatal range, 108–607 μg/dL), progesterone 16.8 nmol/L (elevated relative to male range, 0.5–5.2 nmol/L), and morning cortisol 1.2 μg/dL (low; reference range, 6.2–19.4 μg/dL [4]), suggestive of adrenal insufficiency based on this single measurement. However, serum electrolytes, blood pressure, and pH were all within normal limits. By 1 year and 4 months, ambiguous genitalia persisted, and an ultrasound pelvis (January 2019) reported a structure posterior to the bladder (approximately 1.3 × 0.8 × 1.2 cm) with no visualization of the gonads. Karyotype confirmed a normal 46,XY constitution. Between ages 2 and 3 years, the child sustained multiple low‐trauma fractures–three right tibial and two right clavicular–all healing adequately. Blue sclerae and short stature were documented consistently at each review. At age 6 years, the child presented with multiple fractures since age 3, blue sclerae, short stature, and persistent ambiguous genitalia. There was no history of neonatal jaundice, developmental delay, or previous admission to the intensive care unit, and immunizations were up to date. There was also no history of convulsions, vomiting, polydipsia, polyuria, or maternal drug intake during pregnancy, and family history was non‐contributory with no consanguinity or similar phenotypes reported.

### 2.2 Examination

**FIGURE 1:**
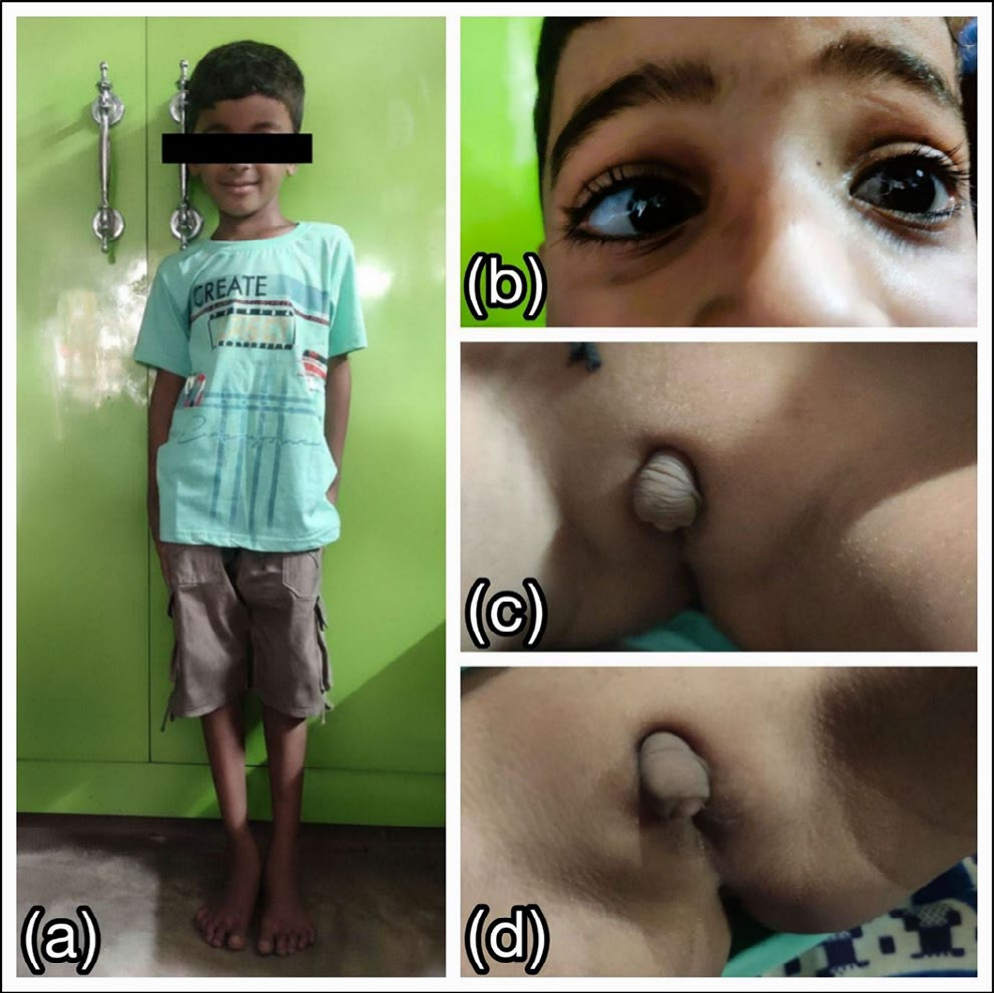
Clinical presentation of the patient. (a) Clinical photograph of the child (age 6 years) showing disproportionate short stature, consistent with osteogenesis imperfecta and growth impairment. (b) Close‐up view of the eyes showing a bluish scleral hue, a classical clinical sign of osteogenesis imperfecta. (c) Local examination of the external genitalia demonstrates micropenis with penile hypospadias and poorly developed scrotum; gonads are not visible nor palpable. (d) Another view of the external genitalia further highlights the findings. All images are arranged clockwise, starting from the top left. All clinical images were obtained with parental consent for academic and publication purposes. The patient's identity has been anonymized in accordance with ethical standards.

Examination revealed a micropenis, penile hypospadias, a poorly formed scrotum, and impalpable gonads. Anthropometry at admission showed height 106 cm, weight 14.2 kg (height *Z*‐score, approximately −3.0; weight *Z*‐score, approximately −2.5, both below the 3rd percentile for age), BMI 12.6 kg/m^2^ (underweight), head circumference 55 cm, upper segment (US) 57 cm, and lower segment (LS) 55 cm (LS < US, disproportionate short stature). No dysmorphic facies or organomegaly were noted. Respiratory, cardiovascular, and neurological examinations were unremarkable. Figure 1 illustrates the key physical examination findings, including disproportionate short stature, a bluish scleral hue, and a micropenis with a poorly developed scrotum.

### 2.3 Investigations

Given the constellation of features, a stepwise multidisciplinary evaluation was undertaken. Ultrasonography performed in 2019, 2023, and 2024 consistently failed to identify testes, instead demonstrating a hypoechoic structure measuring 13 × 11 mm in the left adnexa and a uterine‐like structure measuring 18.5 × 4.5 mm posterior to the bladder. Pelvic magnetic resonance imaging (MRI) in January 2024 confirmed the absence of testicular, ovarian, or prostatic tissue and reported a 15 × 10 mm T2‐isointense to hyperintense structure posterior to the bladder, suggestive of uterine tissue, while hip and sacroiliac joints were normal. Hence, a structure was consistently identified across modalities as a midline, rudimentary organ located posterior to the urinary bladder and anterior to the rectum, measuring approximately 18.5 × 4.5 mm on ultrasonography and 15 × 10 mm on T2‐weighted MRI. The T2‐isointense to hyperintense signal is characteristic of myometrial‐like tissue, and the absence of a clear endometrial stripe or distinct cervical canal further supports the diagnosis of a rudimentary Müllerian remnant rather than a fully formed uterus. Brainstem evoked response audiometry (BERA; December 2023) showed normal waveforms bilaterally, excluding sensorineural hearing loss and syndromic associations such as Perrault syndrome, a rare genetically heterogeneous condition characterized by sensorineural hearing loss and gonadal dysfunction [5]. Endocrine evaluation in January 2024 revealed follicle‐stimulating hormone (FSH) of 16.03 mIU/mL (elevated), luteinizing hormone (LH) of 1.35 mIU/mL (low), testosterone < 7 ng/dL (markedly low), estradiol 13.14 pg/mL, DHEAS < 15 μg/dL, androstenedione < 0.300 ng/mL, and intact parathormone 19 pg/mL, consistent with hypergonadotropic hypogonadism (primary gonadal failure) and adrenal insufficiency. The key clinical findings from birth to diagnosis, along with the current management, have been summarized chronologically in Table 1. Given the pattern of hormonal assays, absence of salt‐wasting crises, adrenal enlargement on imaging, and genetic mutations, congenital adrenal hyperplasia (CAH) was provisionally ruled out by the treating team. These findings prompted further genetic investigation via whole‐exome sequencing. Karyotype was 46,XY, and whole‐exome sequencing performed in June 2024 identified a heterozygous pathogenic frameshift variant in COL1A1 exon 5 (c.441delC; p.Gly148fs*117), confirming osteogenesis imperfecta (autosomal dominant), and a heterozygous variant of uncertain significance (VUS) in CYP11A1 exon 1 (c.119 T > C; p.Ile40Thr), associated with adrenal insufficiency and 46,XY sex reversal. Copy‐number and structural‐variant analysis were negative. Table 2 summarizes the genetic findings, including the genes involved, identified variants, zygosity, classification, reported phenotypes, and their relevance to the present case.

**TABLE 1:** Clinical timeline of events.

Age/Date	Clinical event	Key findings	Action/outcome
Birth (2017)	Term VD, BW 2.7 kg	Ambiguous genitalia noted; no immediate complications	Observation
Day 6	Hormonal assay	Progesterone ↑ (16.8 nmol/L), cortisol ↓ (1.2 μg/dL), DHEAS normal	Suspected adrenal insufficiency and later ruled out
Jan 2019 (1 year 4 months)	USG pelvis	Prostate‐like structure posterior to bladder; uterus/ovaries not visualized (suboptimal)	Baseline imaging
2019	Karyotype	46,XY	Confirmed chromosomal sex
2018–2020 (ages 2–3)	Multiple fractures	5 low‐trauma fractures (3 tibia, 2 clavicle)	Conservative treatment
Dec 2023 (6 years)	Clinical re‐evaluation	Blue sclerae, short stature, micropenis, hypospadias, impalpable gonads	Admission for Endocrine and genetic work‐up
Dec 2023	BERA	Normal bilaterally	Excluded syndromic hearing loss
Jan 2024	MRI pelvis	Uterine‐like structure posterior to the bladder; no testes or ovaries	Consistent with DSD
Jan 2024	Endocrine labs	FSH ↑, LH ↓, testosterone very low, DHEAS/androstenedione undetectable	Suggestive of gonadal dysgenesis and adrenal steroid deficiency
Jan 2024	Whole exome sequencing	COL1A1 pathogenic mutation; CYP11A1 VUS	Dual genetic findings and confirms OI (COL1A1)
2024–ongoing	Management	Zoledronate infusion, Calcium and Vitamin D supplementation; psychosocial and gender counseling	Long‐term multidisciplinary follow‐up

Abbreviations: BERA, brainstem evoked response audiometry; BW, birth weight; COL1A1, Collagen Type I Alpha 1 Chain; DHEAS, dehydroepiandrosterone sulfate; DSD, disorder of sex development; FSH, follicle‐stimulating hormone; LH, luteinizing hormone; OI, osteogenesis Imperfecta; USG, ultrasonography; VD, vaginal delivery; VUS, variant of uncertain significance.

**TABLE 2:** Genetic findings.

Gene	Variant	Zygosity	Classification	Reported phenotype	Relevance to case
COL1A1	c.441delC; p.Gly148fs*117	Heterozygous	Pathogenic	Osteogenesis imperfecta (autosomal dominant)	Explains recurrent fractures, blue sclerae, and short stature
CYP11A1	c.119 T > C; p.Ile40Thr	Heterozygous	Variant of uncertain significance (VUS)	Adrenal insufficiency with 46, XY sex reversal (when biallelic)	Possible modifier explaining ambiguous genitalia and low steroids (unlikely pathogenic)

*Note:* Variant classification according to the American College of Medical Genetics and Genomics (ACMG) guidelines.

### 2.4 Differential Diagnosis

Primary considerations included disorders of androgen biosynthesis (e.g., CYP11A1 defects, CYP17A1 deficiency, 17β‐HSD type 3 deficiency), 46,XY gonadal dysgenesis, ovotesticular DSD, mixed gonadal dysgenesis (45,X/46,XY), persistent Müllerian duct syndrome, androgen insensitivity, and syndromic causes including Perrault syndrome. The differential diagnosis for the skeletal phenotype included osteogenesis imperfecta and syndromic skeletal dysplasias such as campomelic dysplasia (SOX9 mutation).

### 2.5 Treatment

The patient currently receives annual intravenous zoledronate with calcium and vitamin D supplementation to maintain optimal bone mineral density. Caregivers have been counseled about the nature of the skeletal fragility associated with OI and have been advised necessary precautions aimed at ensuring the prevention of future fractures. In addition, multidisciplinary counseling, psychosocial support, and serial monitoring of gonadal development are ongoing. No corrective genital surgery or gonadal biopsy has been undertaken. Genetic counseling has been provided to the family regarding the pathogenic COL1A1 frameshift mutation and the CYP11A1 variant and cascade testing recommended for first‐degree relatives, if feasible.

**FIGURE 2:**
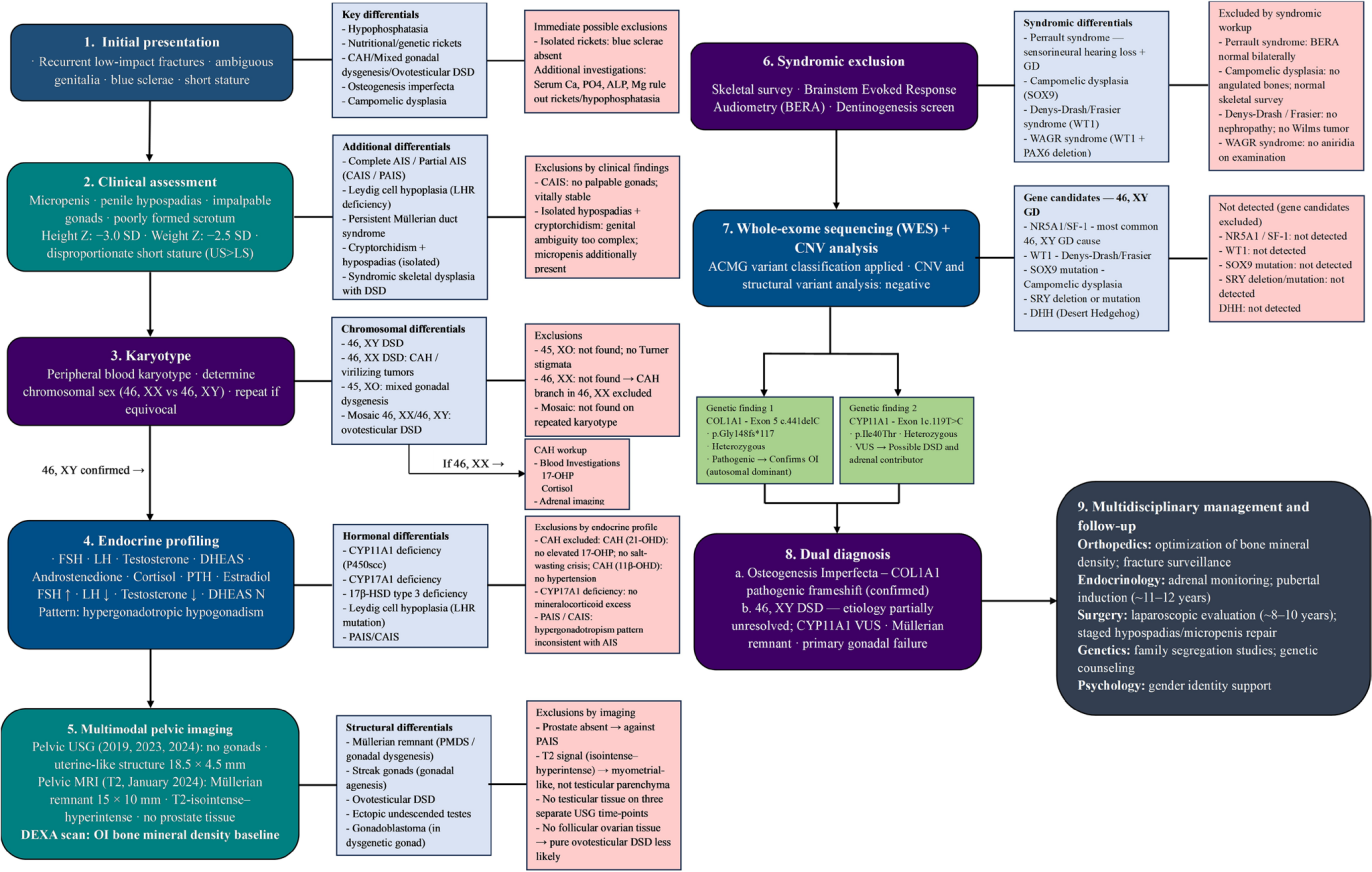
Proposed diagnostic algorithm for the stepwise evaluation of concurrent skeletal dysplasia and disorder of sex development. A stepwise diagnostic flowchart for a child presenting with concurrent recurrent fractures and ambiguous genitalia. Following karyotyping, a 46,XX result triggers a parallel congenital adrenal hyperplasia workup, while a confirmed 46,XY karyotype directs sequential evaluation through endocrine profiling, multimodal pelvic imaging, and syndromic exclusion. Where findings remain inconclusive, whole‐exome sequencing with copy number variant analysis is performed, with results directing to either the OI management pathway (COL1A1 variant) or the DSD management pathway (CYP11A1/DSD‐associated variant). Both pathways converge on multidisciplinary follow‐up. 11β‐OHD, 11β‐hydroxylase deficiency; 17‐OHP, 17‐hydroxyprogesterone; 21‐OHD, 21‐hydroxylase deficiency; ACMG, American College of Medical Genetics and Genomics; AIS, androgen insensitivity syndrome; AMH, anti‐Müllerian hormone; BERA, brainstem evoked response audiometry; CAH, congenital adrenal hyperplasia; CNV, copy number variant; DEXA, dual‐energy X‐ray absorptiometry; DHEAS, dehydroepiandrosterone sulfate; DSD, disorder of sex development; FSH, follicle‐stimulating hormone; GD, gonadal dysgenesis; LH, luteinizing hormone; MRI, magnetic resonance imaging; N, normal; OI, osteogenesis imperfecta; PMDS, persistent Müllerian duct syndrome; US/LS, upper/lower segment ratio; USG, ultrasonography; VUS, variant of uncertain significance; WES, whole‐exome sequencing.

Longitudinal monitoring through routine multidisciplinary follow‐up has been scheduled every six months, with earlier review triggered by surgical concerns, hormonal changes, rapid gonadal size alteration, or emerging gender dysphoria, as gonadal tissue may develop with age, potentially clarifying the underlying etiology of the genital ambiguity. In addition, after multidisciplinary discussion, laparoscopic exploration and reconstructive surgery are being considered around 8–10 years of age, with pubertal induction around 11–12 years are potentially scheduled for the child, and is further elaborated in the following section. Given that this may represent the first reported case of its kind, a proposed diagnostic algorithm for the stepwise evaluation of a child presenting with concurrent skeletal dysplasia and ambiguous genitalia is outlined in Figure 2.

## 3 Discussion

This is, to our knowledge, the first reported case of osteogenesis imperfecta coexisting with ambiguous genitalia in an individual with a 46,XY karyotype. OI is typically caused by COL1A1 mutations and manifests with recurrent fractures, blue sclerae, and short stature [1]. Our patient carried a pathogenic frameshift mutation likely subject to nonsense‐mediated mRNA decay, consistent with the skeletal phenotype [6]. The ambiguous genitalia, however, cannot be explained by OI alone.

The neonatal low cortisol concentration (1.2 μg/dL) and later low androgen precursors raise the possibility of a steroidogenic defect that may have been transient, evolving, or incompletely characterized [4]. Repeated hormonal evaluations, however, did not demonstrate persistently low cortisol concentration; subsequent values remained within the low‐normal range, and the patient exhibited no biochemical or clinical evidence warranting glucocorticoid supplementation. This pattern is suggestive of partial adrenal insufficiency with preservation of basal steroidogenic activity [7]. CYP11A1 (P450scc) deficiency is biologically plausible, as biallelic mutations disrupt cholesterol side‐chain cleavage and lead to adrenal insufficiency with 46,XY sex reversal [8, 9]. However, in this case, only a single heterozygous CYP11A1 VUS was detected (which does not confirm causality). CAH was also excluded on subsequent testing.

No prior clear association between isolated COL1A1‐related OI and ambiguous genitalia in a 46,XY individual emerged from our literature overview. While certain skeletal dysplasias, such as campomelic dysplasia, are known to co‐occur with ambiguous genitalia, a direct pathogenic link between COL1A1 variants and disorders of sex development has not been described [10, 11]. A dedicated search of PubMed, GeneReviews, and curated rare‐disease databases did not identify any previous reports of isolated COL1A1 pathogenic variants causing ambiguous genitalia. Rare syndromic entities combining skeletal anomalies with genital ambiguity exist (e.g., congenital OI‐microcephaly‐cataract syndrome; microcephaly with ambiguous genitalia) [12, 13], but these are genetically and phenotypically distinct. Therefore, the dual phenotype in this patient may represent either two independent conditions—OI from COL1A1 mutation and DSD related to steroidogenic dysfunction, or a novel, as‐yet‐unrecognized syndromic association.

As discussed previously, the long‐term management of this patient necessitates a structured multidisciplinary framework that integrates endocrinology, orthopedics, surgery, genetics, and psychosocial support. From a surgical standpoint, laparoscopic exploration will be potentially planned approximately around the age of 8–10 years, prior to onset of puberty, to locate and evaluate the impalpable gonads; given that dysgenetic gonads in 46,XY individuals carry a heightened risk of gonadoblastoma, a germ cell tumor arising in dysgenetic or undescended gonads, with a reported lifetime risk of up to 15%–30% [14], histologic assessment and potential prophylactic gonadectomy will be considered pending the child's developing gender identity and parental counseling, with a decision expected no later than early adolescence (~age 10–12 years). Management of the Müllerian remnant will be guided by whether it causes symptoms or interferes with future reconstructive surgery. Decisions regarding hypospadias and micropenis repair are deferred until the child can participate in gender identity discussions, typically no earlier than mid‐childhood (8–10 years of age), consistent with current multidisciplinary DSD guidelines [14].

From an endocrinologic standpoint, the heterozygous CYP11A1 VUS warrants ongoing vigilance for partial adrenal insufficiency. Thus, a “stress‐dose” corticosteroid protocol will be considered during physiologic stress, such as surgical procedures or acute illnesses, if clinically indicated. As the patient approaches adolescence, given the prior biochemical evidence of primary gonadal failure (elevated FSH, markedly low testosterone concentrations), pubertal induction at around 11–12 years with exogenous sex steroids will be required, along with gradual dose escalation over 3–4 years to mimic physiological male puberty, subject to the patient'sgender identity trajectory.

Skeletal management will include continued annual zoledronate infusions, along with calcium and vitamin D supplementation, to address COL1A1‐related skeletal fragility and optimize bone mineral density in anticipation of puberty demands. In case of failure of medical therapy in optimization of bone mineral density and prevention of fractures, a decision to undertake prophylactic surgery for the same would be considered by the specialist team. Integrated psychosocial support will accompany the child through gender identity development, the emotional impact of managing two concurrent rare conditions, and the family's adjustment to this complex diagnostic journey.

The case highlights the importance of longitudinal multidisciplinary care; however, a formal claim of a novel syndromic association would require additional reported cases or mechanistic studies, but the present case underscores the need for vigilance in identifying unusual phenotype–phenotype combinations that may expand current genotype–phenotype correlations.

## 4 Conclusion

We describe what is, to our knowledge, an unprecedented association of OI and ambiguous genitalia in a child with a 46,XY karyotype. The patient is a 6‐year‐old child with genetically confirmed COL1A1‐related OI and lifelong ambiguous genitalia. Multiple imaging studies suggested a small uterine‐like structure but revealed no palpable or imaging‐confirmed testicular tissue. Whole‐exome sequencing confirmed a pathogenic COL1A1 frameshift mutation explaining the OI phenotype and identified a heterozygous CYP11A1 variant of uncertain significance, although no other definitive genetic cause for the DSD was detected. While the OI phenotype was clearly explained, the ambiguous genitalia remain unresolved, with a possible but unproven contribution of CYP11A1. This case may represent either the coincidental occurrence of two rare conditions or a previously unrecognized association. This index case expands the phenotypic spectrum of OI‐associated presentations and highlights the value of comprehensive genomic and endocrine evaluation in atypical cases. Longitudinal multidisciplinary follow‐up is essential to clarify the underlying etiology and guide management.

## Author Contributions

**Harshita Agarwal:** conceptualization, data curation, investigation, methodology, resources, writing – original draft, writing – review and editing. **Tathagata Jha:** conceptualization, formal analysis, resources, validation, visualization, writing – original draft. **Mohammad Orooj Azmi:** conceptualization, data curation, formal analysis, investigation, methodology, resources, writing – original draft. **Soumita Sarkar:** conceptualization, investigation, project administration, supervision, visualization, writing – review and editing. **Vinay Suresh:** methodology, resources, supervision, writing – review and editing.

## Funding

The authors have nothing to report.

## Consent

The authors obtained written informed consent from the patient's guardians for genetic testing, anonymized use of clinical data, and publication of this case report (including images), with all identifying details removed.

## Conflicts of Interest

The authors declare no conflicts of interest.

## Data Availability

Data sharing is not applicable as no datasets were generated or analyzed for this case report.
